# Diet in Disease

**Published:** 1892-10-22

**Authors:** Ernest Hart

**Affiliations:** Bachelier-ès-Sciences-es-Lettres (rèstreint), formerly Student of the Faculty of Medicine of Paris, and of the London School of Medicine for Women


### Diet in Disease.

By Mrs. Ernest Hart, Bachelier-ès-Sciences-es-Lettres (rèstreint), formerly Student of the Faculty of Medicine of Paris, and of the London School of Medicine for Women.

### IV.—STIMULANTS (*continued*).

### The Coca of Peru—Coca Wine.

That all men and women feel the weariness of life is testified by the fact that the people of all nations and climes have the universal habit of daily seeking a restorative and stimulant in one of the vegetable products which contain a substance or alkaloid capable of exercising a definite effect on the nervous and cardiac systems. Thus, the Chinese and Japanese sip their tea, and the English, following their example, brew the five o'clock cup of the fragrant herb to sustain them in the day's work; the Arab and Turk seek, like the French and Germans, restorative powers in the aromatic coffee berry; the Cingalese chew the betel nut; and the natives of Peru on the slopes of the Andes find in coca leaves a principle which sustains the body in fatigue and comforts the mind in hopelessness. Von Bibra says of coca: "It satisfies the hungry, lends new strength to the weary and fatigued, and makes the unhappy forget his grief." What, then, is this strange substance which seems to conceal a fairy's wand? We shall find, however, that, resembling other fairies' wands for the cure of the plagues of life, it may turn, like the magician's rod, into a viper.

**Coca is obtained from the leaves of a shrub-like plant called the Erythroxylon Coca.** It is a native of Peru and Bolivia, where it has been cultivated with the greatest care from the remotest antiquity. When Peru was conquered by the Spaniards in the sixteenth century, and the ancient and interesting people of this country were discovered, it was found that their Incas or kings looked upon the cultivation of the coca plantations as a public and national duty, and also that the strange custom prevailed among the Peruvians of chewing the leaves of the coca plant during frequent and short periods of repose, specially set aside for this purpose. This custom prevails to this day among the half-bred Indians of Peru and Bolivia. Three or four times a day the Indian, labouring in the mines or on the plantations, retires from work, and, lying down in a comfortable position, he draws from a leather pouch a few leaves of coca; these he rolls into a ball, which he puts into his mouth and slowly chews, adding from time to time a small amount of unslaked lime, which is said to bring out the true flavour of the leaf. The chewing causes a copious flow of saliva, which is swallowed, but the masticated leaves are rejected when all they contain has been thoroughly extracted. The Indian of the Andes is by nature gloomy, taciturn, and melancholy, but when chewing the coca leaf in repose he seems to be in a condition of passive happiness, and for a short time to be removed from the depressing effects of the toil, the poverty, and the hardship of his lot. The indulgence in coca may be pushed to the extent of intoxication, or the habit of chewing may enslave a man like that of chewing, but these cases are not common among the Indians.

**The Physical Effects of Coca** are, however, more salutary, and in many respects more remarkable than the mental. It is universally acknowledged that coca stills hunger, overcomes drowsiness, and increases bodily activity. All travellers in the Andes bear testimony to the wonderful power shown by the Indians of enduring fatigue, cold, wet, and exposure, with only the scantiest allowance of poor food, if they are supplied with coca. The life of the Indian of the Andes is one of extraordinary toil and hardship. His diet consists mainly of a small quantity of maize and frost-dried potatoes; he is constantly exposed to the intense heat of the plains or to the terrible cold of the high plateaus of the Cordilleras; the toil exacted of him in the mines, and the plantations, is excessive, but he is yet able to perform, not exceptionally, but constantly, and as a matter of daily life, the most astonishing feats of endurance on a diet which would be absolute starvation to a European, or even for a time without food at all, by the aid of the power which coca gives. Von Tschudi, the naturalist and traveller, gives a remarkable account of an Indian, 62 years of age, who was employed by him in digging for five days and nights, with only an interval of sleep of two hours each night. During this time it is asserted that he never tasted food, though at intervals of from two to three hours he chewed half an ounce of coca leaves. The work done, he accompanied Von Tschudi on foot a two days' journey of seventy miles across the level heights, halting only to chew his coca. The most striking stories are told of the men engaged in the postal service, who, half naked, traverse the icy slopes of the Andes carrying the heavy mail bag. These men walk from 200 to 300 miles, crossing the mountain by paths rising 13,000 and 14,000 feet above the sea level; their scanty clothing is an ill protection against the fierce snowstorms, the intense cold, and the rarefied air of the Andes. Their food for the journey consists of from one to two pounds of dried maize and potatoes, but if supplied with sufficient coca to chew they endure cold, hunger, fatigue, and sleeplessness not only without complaint, but without even seeming to be aware of them. In like manner the Indian labourers in the mines of the Cordilleras, whose toil is spoken of as incessant and excessive, and performed in damp, cold, and darkness; the shepherds tending their flocks of alpacas on the bleak Pampas, and the farmers irrigating their fields at night in mid-winter on the high plateaus, standing often knee-deep in icy water and exposed to cutting blasts, are all said to be equally inured to a life of surprising hardship and privation by the daily use of coca. It is stated, however, that though no hermit or monk ever lived so ascetic a life as these poor Indians, yet the appetite for food is only stayed not destroyed by coca, and that if anyone is kind and generous enough to feed them, they eat with voracity and evident enjoyment. It is also stated that if they change their food and give up coca they lose their power of endurance; and, moreover, that the Spaniards who go to work in the mines cannot stand the great hardships of the life and the inclemency of the Cordilleras till they take to the regular use of coca. Von Tschudi tells us that this life of silent endurance and bitter abnegation may be much prolonged even in one instance to 130 years.

**The other remarkable effect of Coca is the influence it exercises in the respiratory centres,** so that the rarefied air of the higher altitudes of the Andes can be breathed without the distress usually occasioned at the height of thirteen or fourteen thousand feet above the sea. All travellers speak of the extraordinary way the Indian porters will keep up with the quick pace of the mule along the roughest mountain paths without ever showing any signs of breathlessness.

Though wonderful but little credited stories were told for two centuries of the staying powers of coca leaves, no attempt was made to introduce them into Europe and scientifically to test their value either as dietetic or therapeutic agents till about forty years ago. The first experiments were nugatory, as the substances which give the leaves their subtle power escaped or were volatilised during the voyage. Care has, however, since been taken to enclose the leaves in air-tight boxes, with the result that a great variety of fluid extracts and wines of coca are now made, and are widely recommended for their tonic properties.

I have had some small experience of the value of one of these under exceptional circumstances. When reading some years ago in Paris, under pressure of time, for public *viva voce* medical examinations, I found th-t I could work for from fourteen to sixteen hours a day, for three or four weeks together (work ending in successful examinations) without mental or physical fatigue, or bad after-results if I took a small daily dose of coca wine. My modicum was a bottle a week, discontinued immediately my task was done. Recently, when making a long convalescence from influenza, in which depressing cardiac symptoms were marked, I found again in coca a good and reliable restorative. I have no reluctance in saying that if I had to accomplish some severe work which drew exhaustingly on my full mental and physical powers—such as preparing in a short time for a public scientific examination in a foreign language, doing literary work under pressure, or nursing one dear to me through a serious illness—I would unhesitatingly, and with good conscience, seek the support and power of endurance coca can give.

Mr. Eber Caudwell published an interesting account in the *British Medical Journal* (vol. i. p. 17, 1888) of the effect coca had on himself in enabling him to go through long hours of toil without sleep, and while preserving his full mental activity and vigour. When the pedestrian Weston was performing his feats of walking, it was noticed that he was always chewing a greenish substance, and after repeated inquiries he at length admitted that this was coca, to which he trusted to maintain his muscular activity without fatigue. Singers find that coca enables them to inspire more deeply, and to hold their breath longer than they could otherwise do.

**But Coca is not without danger**; and what to the poor Indian may suffice for food; what may give to the student the hours of sleep for study without increasing fatigue; what may yield to the traveller and labourer the power of accomplishment without inducing weariness, may become to the fashionable lady another source of self-indulgence. As a rule, all well-to-do persons are over-nourished, and the debility of which they frequently complain is not due to the want of food and stimulants, but to having fatigued and broken down the bodily machine in its effort to digest and get rid of the amount of food, wine, and "strengthening substances" taken. The only justification for adding coca to one's daily diet would be an undue amount of labour to be undertaken, or the lessening of the usual amount of food taken. Coca gives to the Indian the power to endure a life of penurious toil and privation; in like manner a rigid ascetism should with us accompany the use of coca. The knowledge of its powers cannot fail, however, to be of value to the pedestrian, the traveller, and to those who work hard and live low.

**Cocaine.**—The most valuable substance which has been extracted from coca is cocaine, which from its power of producing local and superficial anæsthesia has been much used of recent years in operations on the eyes, teeth, &c. Cocaine rapidly became a fashionable nerve stimulant and sedative, but its use has been accompanied with many fatal accidents, and with the introduction of a new disease, namely, a morbid longing for cocaine, or *cocainism.* Thus the fairy's wand, that stayed the hunger of the poor and enduring Indian in the Andes, has become a viper in the bosom of a self-indulgent society. The sale of cocaine to the public, other than as a drug, has been forbidden in France, and will probably also be rendered illegal in America.

**The Scientific Study of Cocaine** has led to a better comprehension of the mysterious qualities of the coca leaf. The first effect is sedative, rapidly followed by stimulation, in which the heart beats are quickened, the nervous system becomes more active, the intelligence more acute, and the muscles pass more easily into a state of contraction. Dr. Mantegazza says that when he was under the influence of coca he had an irresistible inclination to gymnastic exercise. The absence of the sense of hunger seems to be due not only to the anæsthetic effect of the cocaine on the nerve ends of the stomach, but also to the fact that coca is an actual economiser of food, and so modifies the vital processes in muscle as to effect its chemical activity, and renders it capable of performing an equal and greater amount of work with a lesser consumption of carbo-hydrates (Stockman). The absence of emaciation or subsequent debility or other bad results after the most exalted powers of the organism have been called forth, point to coca being more than a nerve stimulant, but also an actual economiser of the bodily expenditure. If it diminishes the consumption of carbo-hydrates during muscular activity, that is to say, enables the machine to work with less fuel, less oxygen will be required, and hence is explained the effect coca has in preventing breathlessness when ascending high mountains. Excessive quantities of coca cause headache, giddiness, mental aberration, and ultimately nervous breakdown.

